# Early Exposure of Kidney Transplant Recipients with Chronic Antibody-Mediated Rejection to Tocilizumab—A Preliminary Study

**DOI:** 10.3390/jcm12227141

**Published:** 2023-11-17

**Authors:** Capucine Arrivé, Marvin Jacquet, Elodie Gautier-Veyret, Thomas Jouve, Johan Noble, Dorothée Lombardo, Lionel Rostaing, Françoise Stanke-Labesque

**Affiliations:** 1 Laboratory of Pharmacology, Pharmacogenetics and Toxicology, Grenoble Alpes University Hospital, 38043 Grenoble, France; carrive1@chu-grenoble.fr (C.A.);; 2 University Grenoble Alpes, Inserm, CHU Grenoble Alpes, HP2, 38000 Grenoble, France; 3 Department of Nephrology, Dialysis, Apheresis and Transplantation, Grenoble Alpes University Hospital, 38043 Grenoble, France; 4 Department of Pharmacy, Grenoble Alpes University Hospital, 38043 Grenoble, France

**Keywords:** tocilizumab, pharmacokinetics, antibody-mediated rejection, kidney transplant

## Abstract

Tocilizumab prevents clinical worsening of chronic antibody-mediated rejection (CAMR) of kidney transplant recipients. Optimization of this treatment is necessary. We identified the determinants of early tocilizumab exposure (within the first three months) and investigated the relationship between early plasma tocilizumab exposure and graft function. Patients with CAMR who started treatment with tocilizumab were retrospectively included. Demographic, clinical, and biological determinants of the tocilizumab trough concentration (C_min_) were studied using a linear mixed effect model, and the association between early exposure to tocilizumab (expressed as the sum of C_min_ over the three first months (M) of treatment (ΣC_min_)) and the urinary albumin-to-creatinine ratio (ACR) determined at M3 and M6 were investigated. Urinary tocilizumab was also measured in seven additional patients. Seventeen patients with 51 tocilizumab C_min_ determinations were included. In the multivariate analysis, the ACR and time after tocilizumab initiation were independently associated with the tocilizumab C_min_. The ΣC_min_ was significantly lower (*p* = 0.014) for patients with an ACR > 30 mg/mmol at M3 and M6 than for patients with an ACR < 30 mg/mmol. Tocilizumab was detected in urine in only 1/7 patients. This study is the first to suggest that early exposure to tocilizumab may be associated with macroalbuminuria within the first six months in CAMR patients.

## 1. Introduction

Chronic antibody-mediated rejection (CAMR) is a major complication of kidney transplantation, leading to degradation of renal function and, ultimately, loss of the graft. This complication is observed in up to 5% of first kidney transplant recipients [1,2]. Treatment of CAMR remains a challenge as there is no current consensus on its management, and the treatment options are limited beyond optimization of immunosuppression [3]. Intravenous administration of immunoglobulins, apheresis, and pulse of corticosteroids are a part of the treatment strategies in association or not with biologics disrupting B-cells (rituximab), complement (eculizumab) or interleukin 6 (IL-6) pathways (tocilizumab TCZ) [4]. Indeed, some recent studies suggested that targeting IL-6 pathway in particular could be a promising pharmacological strategy in the management of CAMR, given the key role of IL-6 in the regulation of systemic inflammation, the development and maturation of T cells and B cells leading to the synthesis of donor-specific antibody (DSA) [5,6,7,8,9].

TCZ is a humanized immunoglobulin G (IgG) 1 monoclonal antibody that competitively inhibits the IL-6 signaling pathway by binding to both its soluble and membrane-bound receptors [10]. TCZ is approved for treatment of rheumatic diseases (rheumatoid arthritis and idiopathic juvenile arthritis), in which it normalizes C reactive protein (CRP) levels and erythrocyte sedimentation rates within two weeks [11]. Recent studies suggested that TCZ could also be a promising therapeutic for the salvage treatment of CAMR [12], as six months of TCZ treatment reduced microvascular graft inflammation [13] and stabilized renal function [12,13,14,15,16,17,18,19,20]. In patients with CAMR, TCZ is administrated intravenously, monthly, at a dose of 8 mg/kg (with a maximum dose of 800 mg), in combination with corticosteroids and mycophenolate mofetil or an anticalcineurin, or belatacept. TCZ presents highly variable plasma concentrations in patients with rheumatic diseases [21,22] and kidney-transplant candidate patients undergoing desensitization [23]. Several studies even suggested a link between the TCZ concentration and clinical efficacy [11,24,25] in patients with rheumatic diseases. However, the pharmacokinetics of TCZ have never been studied for patients with kidney CAMR. Moreover, as for numerous other monoclonal therapeutic antibodies, it can take up to five to eight weeks to reach the pharmacokinetic steady state due to its long elimination half-life. Given the clinical severity of CAMR and the high cost of TCZ, the study of a potential association between early exposure to TCZ and kidney function is therefore of major importance.

The aims of this study were to describe the variability of TCZ early exposure, i.e., within the first three months (M) of treatment, identify the determinants of this variability, and investigate the relationship between early plasma TCZ exposure and graft function.

## 2. Materials and Methods

### 2.1. Study Design

We performed a retrospective monocentric cohort study that was approved by the Grenoble University Hospital review board (registration RnIPH 2022, protocol TOCIREJET; CNIL number: 2205066 v 0). This study was conducted according to the ethical guidelines of the Declaration of Helsinki [26]. As we previously showed that TCZ concentrations are highly variable among kidney-transplant candidates [23], the TCZ trough concentrations (C_min_) of all patients treated with TCZ were monitored during their routine care.

The inclusion criteria were being a renal-transplant patient who started TCZ treatment (intravenous 8 mg/kg every 4 weeks) as first-line salvage therapy for CAMR in the Nephrology Unit of the Grenoble University Hospital between December 2020 and May 2022, with TCZ plasma C_min_ available at M1, M2, and M3 after the initiation of treatment. CAMR was defined according to last Banff classification [27] as the presence of chronic transplant glomerulopathy (cg score > 0) with severe peritubular capillaries (ptc score), basement membrane multilayering either with or without C4d deposition in peritubular capillaries, and the presence of anti-HLA DSA. In case of the association with at least moderate microvascular inflammation (g + ptc ≥ 2), lesions were defined as CAMR. The exclusion criteria were previous TCZ treatment or treatment for any indication other than CAMR.

The following data were retrospectively collected from the medical records at M1, M2, and M3: sex, age, weight, body mass index (BMI), treatment administration (dose, TCZ initiation date, time since the first dose, immunosuppressive co-treatment), and biological data (TCZ C_min_, serum creatinine, aspartate aminotransferase [ASAT], alanine aminotransferase [ALAT], gamma-glutamyl-transferase [GGT], total protein, and CRP levels, CKD-EPI glomerular filtration rate [GFR], and the urinary albumin-to-creatinine ratio [ACR]). The ACR was also collected at M0 (the day of TCZ initiation treatment) and at M6 (6 months after TCZ initiation treatment).

The biological parameters were measured on samples taken the same day as the samples used for the determination of the TCZ concentration. A patient was considered to be macroalbuminuric when his/her ACR was > 30 mg/mmol [28,29]. Liver function was considered to be impaired when transaminases were greater than three times the upper limit of normal. Finally, the urinary excretion of TCZ was evaluated in a separate cohort of seven patients (see Supplementary Materials Table S1).

### 2.2. Measurement of TCZ Concentration and Exposure

TCZ C_min_ were determined at months M1, M2, and M3 after TCZ initiation using a validated liquid chromatography-tandem mass spectrometry method, as previously described [30]. The imprecision of this method was < 10%, with a bias < 15% over the validated range of 1.0–200.0 mg/L.

Early exposure to TCZ is expressed as the sum of the three C_min_ (ΣC_min_) over the first three months of treatment for a given patient.

### 2.3. Statistical Analysis

Continuous data are expressed as medians (10th–90th percentile) and categorical variables as percentages (numbers). The relationship between the TCZ C_min_ (dependent variable) and other variables (age, gender, time after first administration, dose, weight, BMI, serum creatinine, GFR, urinary ACR, total serum protein, and co-treatment with tacrolimus, mycophenolic acid, or belatacept) was tested using linear mixed-effect models with the patient and number of injections as random factors (to account for the multiplicity of TCZ C_min_ values obtained for the same patient at different times post injections). Multivariate linear mixed-effect analysis including all factors and covariates with a *p*-value < 0.15 in univariate analyses was conducted.

A Friedman test was used to compare the ACR between months M0, M2, M1, M3, and M6. The association between early TCZ exposure (ΣC_min_) and the ACR determined at M3 and M6 was investigated using the Mann–Whitney test and Spearman *t* test.

The Shapiro–Wilk test was used to assess the normality of the distribution of continuous variables, and Levene’s test was used to assess the homogeneity of the variances. Data were log-transformed to satisfy the conditions of application of linear models when not normally distributed. All statistical tests were performed with an alpha threshold of 0.05. Statistical tests were performed using Jamovi^®^ (version 1.6, Sydney, Australia).

## 3. Results

### 3.1. Baseline Characteristics

**Figure 1:**
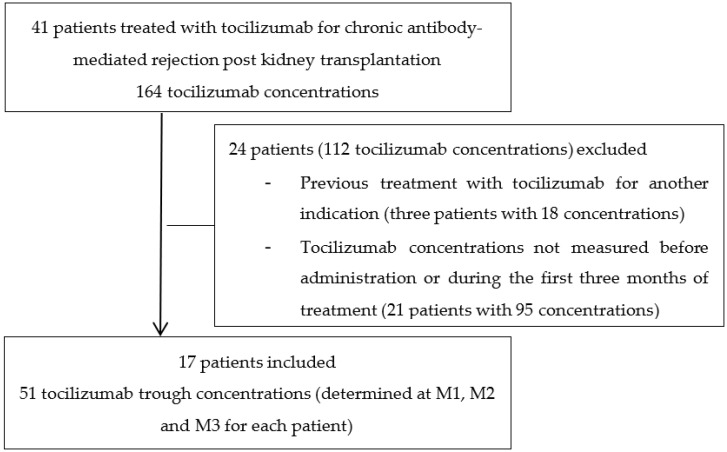
Flow chart of this study.

Seventeen kidney transplant patients treated with TCZ for CAMR were included, (see flow chart in Figure 1). Ten (58.8%) were men. Their median (10th–90th percentile) age, weight, and BMI were 52 (37–73) years, 75 (53–85) kg and 23.3 (20.4–29.3) kg/m^2^, respectively. All patients (100%) were treated with corticosteroids, fourteen (82.4%) received tacrolimus, fifteen (88.2%) received mycophenolic acid, and four (23.5%) received belatacept. The biological characteristics of the patients at M1, M2 and M3 are presented in Table 1. Regarding the histological data, the median g+cpt score before TCZ was 4 (3–5), indicating CAMR. Median cg score was 2 (1–3).

### 3.2. Variability of TCZ trough Concentrations and Early Exposure (ΣC_min_)

The TCZ C_min_ at M1, M2, and M3 are described in Table 1. The median (10th–90th percentile) of all TCZ C_min_ was 19.7 mg/L (7.7–36.6) and the coefficient of variation was 42.2%. The median (10th–90th percentile) of ΣC_min_ was 57.6 mg/L (26.2–100.0) and the coefficient of variation was 38.3%.

### 3.3. Determinants of the TCZ trough Concentration

In the univariate analysis, time from treatment initiation, co-treatment by tacrolimus, and the ACR were associated with the TCZ C_min_ (Table 2). In the multivariate analysis, only the ACR and time from TCZ initiation remained independently associated with the TCZ C_min_.

### 3.4. Association between Early TCZ Plasma Exposure and Kidney Function

**Figure 2:**
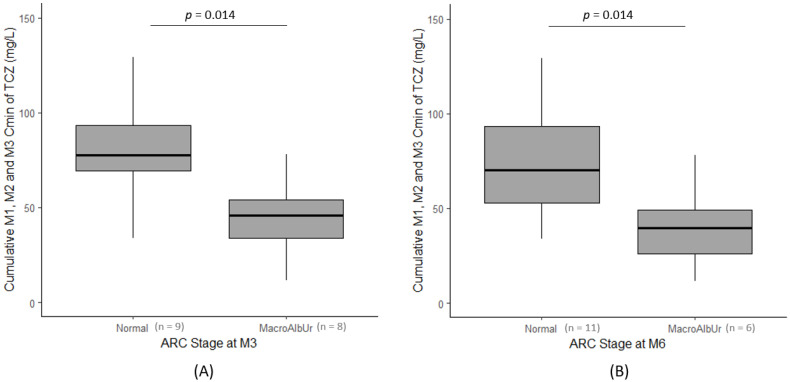
Cumulative M1, M2, and M3 C_min_ of TCZ in patients with an ACR < 30 mg/mmol (normal) or > 30 mg/mmol (macroalbuminuria (MacroAlbUr)) at M3 (**A**) and M6 (**B**). Boxplots represent the medians and 25th–75th percentile.

The ΣC_min_ were significantly lower (*p* = 0.014) for patients with ACR > 30 mg/mmol at M3 (median ΣC_min_ of 46.0 mg/L [21.9–63.8]) than for patients with ACR < 30 mg/mmol (median ΣC_min_ of 77.7 mg/L [45.8–106.0]) (Figure 2A).

At M6, the ΣC_min_ were also significantly lower (*p* = 0.014) for patients with ACR>30 mg/mmol (median ΣC_min_ of 39.9 mg/L [19.0–63.5]) than for patients with ACR < 30 mg/mmol (median ΣC_min_ of 70.2 mg/L [48.8–100.0]) (Figure 2B). Conversely, the ΣC_min_ were not associated with the glomerular filtration rate (*p* = 0.891).

Figure S1 presents the temporal evolution of the ACR before the TCZ administration (M0) and at M1, M2, M3, and M6 after the initiation of the TCZ treatment. All patients who were macroalbuminuric at M0 (two missing data points) kept the ACR > 30 mg/mmol at M1, M2, M3, and M6 (except one patient with an ACR = 21.1 mg/mmol at M6) post TCZ treatment initiation. Except one, all patients who had an ACR < 30 mg/mmol at M0 kept a normal ACR at M1, M2, M3, and M6. The ACR values were not significantly different between months (*p* = 0.430).

As urinary loss of the monoclonal antibody rituximab was previously described in patients with kidney disease, such as those with the nephrotic syndrome [31,32], we investigated whether urinary excretion of TCZ could contribute to the variability of the TCZ plasma concentrations. We measured the TCZ urinary concentrations in a separate cohort of seven additional kidney transplant patients with CAMR who were treated with TCZ. The characteristics of this separate cohort are presented in the Supplementary Materials (Table S1). For 6/7 patients, the urinary concentration of TCZ was lower than 1 mg/L, whereas their urinary ACRs ranged from 10 to 343.9 mg/mmol. The only patient who had detectable TCZ in urine (2.9 mg/L) also presented with the highest ACR of the study population (ACR = 948 mg/mmol).

## 4. Discussion

This study is the first to describe the pharmacokinetics of TCZ in kidney transplant patients with CAMR. The TCZ C_min_ showed high interindividual variability among the CAMR patients, as recently reported in a separate cohort of kidney-transplant candidates undergoing desensitization [23]. These data highlight the need to identify the determinants of the variability of the TCZ C_min_ to individually adjust the dose of TCZ.

Our results showed that time since TCZ initiation was an independent determinant of the TCZ C_min_ in the multivariate analysis. The impact of time since TCZ initiation on the TCZ C_min_ is consistent with the long elimination half-life of TCZ, which requires up to three to four months of treatment to reach the pharmacokinetic steady state equilibrium. The ACR was also an independent determinant of TCZ C_min_. Moreover, early TCZ C_min_ measured within the first three months were higher for patients with an ACR < 30 mg/mmol than for those with an ACR > 30 mg/mmol.

Conversely, the TCZ C_min_ were not associated with the glomerular filtration rate. These data were expected since, in the absence of nephropathy, the clearance of monoclonal antibodies involves target-mediated drug disposition and non-specific proteolytic elimination but not glomerular filtration, given their high molecular weight.

However, the urinary excretion of monoclonal antibodies has been reported in certain particular clinical settings, including nephropathy. For example, patients with membranous nephropathy, such as nephrotic syndrome, showed urinary loss of rituximab and insufficient drug exposure [31,32], requiring higher doses or more frequent administrations [33,34]. Urinary excretion of rituximab appeared to be dependent on the severity of proteinuria, as urinary loss of rituximab was observed in high proteinuria (ACR of 16,469 mg albumin/g creatinine) in a case report of two patients with nephrotic syndrome [35]. Interestingly, rituximab excretion in urine appeared to be associated with IgG urinary excretion, suggesting that urinary monoclonal antibody excretion may occur when the glomerular filter loses selectivity [35]. Although CAMR is a different clinical setting of kidney disease, we wished to further explore whether urinary loss of TCZ could contribute to the variability of the TCZ C_min_. In a separate cohort of kidney transplant patients with CAMR, urinary TCZ concentrations were below the limit of quantification (1 mg/L) for 6/7 patients, for whom the ACR ranged from 10 to 343.9 mg/mmol. The only patient who had detectable TCZ in urine (2.9 mg/L) also had the highest ACR of this study’s population: three-fold higher than that of other patients (see Supplementary Materials). Such low urinary excretion (in comparison to plasma concentrations) with such a high urinary ACR suggests that the urinary excretion of TCZ did not contribute to the variability of TCZ plasma C_min_ observed in our cohort of 17 patients in whom the ACR ranged from 0.3 to 666.7 mg/mmol. Urinary excretion of monoclonal antibodies may occur in patients with extremely severe proteinuria, as reported for TCZ in the present study and as previously reported for rituximab [35], but this result must to be confirmed in larger cohorts.

Patients with chronic kidney disease and albuminuria present elevated biomarkers of inflammation [36]. In the Framingham Offspring Cohort, higher TNF-*α*, IL-6, and TNF receptor 2 levels were associated with the ACR [37]. The elimination of TCZ depends on binding to its target and therefore increases when the number of IL-6 receptor increases [38]. In our study, inflammation (accessed by CRP levels) could not be studied as a determinant of early TCZ exposure because all patients had undetectable CRP. This result was expected because CRP production rapidly decreases with TCZ treatment [11]. Further pharmacokinetic/pharmacodynamic (PK/PD) studies investigating the impact of albuminuria on TCZ clearance are required to investigate the underlying mechanisms associated with reduced TCZ plasma concentration in patients with macroalbuminuria, and to understand whether target-mediated drug disposition could contribute to the increased clearance of TCZ in patients with macroalbuminuria, as shown in the present study.

The description of the determinants of the variability of TCZ exposure is the first necessary step to consider the possibility of future personalization of the dose of TCZ. Additional clinical data linking TCZ plasma exposure and clinical efficacy and safety in kidney transplant patients with CAMR are required to determine the target therapeutic concentration range and then personalize the TCZ dosage according to the determinants of its variability. However, since the ACR presented weak variability throughout TCZ treatment courses (present study), our observations suggested that dose adjustment could be made from the first dose of TCZ according to the presence or the absence of a macroalbumuria, and not only according to the body weight of the patient.

The retrospective and monocentric design of our study and the limited sample size of our cohort were the main limitations of the present paper. However, our data, although preliminary, are the first to show that early exposure to TCZ may be associated with macroalbuminuria in the CAMR patients. In addition to the body weight, albuminuria could be a parameter to take into account to individualize the TCZ dosage. Further prospective and multicentric studies on larger cohorts should be performed to confirm our results and determine a threshold for early TCZ exposure associated with a clinical response in terms of efficacy and safety.

## Figures and Tables

**Table 1:** Characteristics of tocilizumab therapeutic drug monitoring and laboratory parameters throughout the follow up (months (M) M1, M2 and M3).

	M1 (*n* = 17)	M2 (*n* = 17)	M3 (*n* = 17)
Tocilizumab pharmacological data			
Trough concentration (mg/L)	17.4 (6.7–24.6) CV = 38.6%	18.7 (11.0–31.2) CV = 41.1%	20.3 (8.8–39.2) CV = 45.0%
Dose (mg)	600 (426–694)	600 (434–669)	600 (392–683)
Laboratory parameters			
Creatinine (µmol/L)	151 (112–190)	150 (113–190)	147 (108–196)
GFR (mL/mn/1.73 m^2^)	44 (30–55)	41 (27–61)	40 (29–60)
ACR (mg/mmol)	9.3 (1.1–160)	10.2 (0.9–149)	8.3 (0.9–100)
Total proteins (g/L)	68 (64–71)	69 (60–72)	67 (62–72)
Hepatic function altered ^a^ % (*n*)	6.3 (1)	6.3 (1)	6.3 (1)
C reactive protein (mg/L)	<4 (<4–<4)	<4 (<4–<4)	<4 (<4–<4)

Data are presented as medians (10th–90th percentile) or percentages (*n*); GFR, CKD-EPI glomerular filtration rate; ACR, urinary albumin-to-creatinine ratio; CV, coefficient of variation. ^a^ Liver function is considered impaired if transaminases are greater than three times the upper limit of normal.

**Table 2:** Univariate and multivariate linear mixed-effect regression analyses for the identification of the determinants of log tocilizumab trough concentrations (*n* = 51) during longitudinal therapeutic drug monitoring.

Variable	Univariate Analysis			Multivariate Analysis		
	Estimate	95% Confidence Interval	*p*-Value	Estimate	95% Confidence Interval	*p*-Value
log(Age)	−0.34	−1.5–0.82	0.578			
Gender (male–female)	0.16	−0.10–0.41	0.253			
log(Time after TCZ initiation)	0.40	0.18–0.61	0.001	0.42	0.17–0.66	0.002
log(Dose)	0.96	−0.094–2.0	0.087	0.88	−0.0044–1.8	0.067
Weight	0.0053	−0.0023–0.013	0.188			
log(BMI)	0.57	−1.1–2.2	0.507			
Creatinine	0.00030	−0.0036–0.0030	0.857			
GFR	0.0014	−0.0072–0.0099	0.758			
log(ACR)	−0.13	−0.24–−0.020	0.037	−0.11	−0.21–−0.0085	0.050
Total protein	−0.0098	−0.029–0.0092	0.316			
Tacrolimus (yes–no)	0.33	0.033–0.64	0.046	0.22	−0.30–0.47	0.107
Mycophenolic acid (yes–no)	−0.084	−0.49–0.32	0.690			
Belatacept (yes–no)	0.057	−0.25–0.37	0.725			

TCZ, tocilizumab; BMI, body mass index; GFR, glomerular filtration rate; ACR, urinary albumin-to-creatinine ratio. Significant values in bold.

## Data Availability

The data that support the findings of this study are available from the corresponding author upon reasonable request.

## Supplementary Materials

The following supporting information can be downloaded at https://www.mdpi.com/article/10.3390/jcm12227141/s1, Table S1: Characteristics of seven patients with simultaneous tocilizumab measurement in the blood and urine. Figure S1: Temporal evolution of the urinary albumin-to-creatinine ratio before tocilizumab administration (M0) and at M1, M2, M3 and M6 after tocilizumab administration.

## Abbreviations

ALAT	alanine aminotransferase
ASAT	aspartate aminotransferase
ACR	urinary albumin-to-creatinine ratio
BMI	body mass index
BSA	body surface area
CAMR	chronic antibody-mediated rejection
C_min_	trough concentration
ΣC_min_	sum of the three C_min_ measurements over the three first months of treatment
CV	coefficient of variation
DSA	donor-specific antibody
GFR	glomerular filtration rate
IgG	immunoglobulin G
IL-6	interleukin 6
M	month
TCZ	tocilizumab
