# Supplementary material for: Early Exposure of Kidney Transplant Recipients with Chronic Antibody-Mediated Rejection to Tocilizumab—A Preliminary Study
Source: J Clin Med. 2023 Nov 17;12(22):7141. doi: 10.3390/jcm12227141 (PMC10672331; doi:10.3390/jcm12227141)
Supplement: Supplementary file 1 [file jcm-12-07141-s001.zip › jcm-2639276-supplementary.pdf]

Journal of Clinical Medicine

Table S1. Characteristics of seven patients with simultaneous tocilizumab measurements in the blood and urine.

	Age (years)	Sex	Dose (mg)	Route of administration	TCZ plasma concentration (mg/L)	TCZ urinary concentration (mg/L)	ACR (mg/mmol)	GFR (mL/mn/1.73m²)
1	41	Male	162	SC	67.9	<1	/	31
2	73	Male	552	IV	37.5	<1	10	39
3	54	Male	736	IV	40.5	<1	41.8	36
4	31	Male	162	SC	75.8	<1	67.5	/
5	61	Female	480	IV	36.2	<1	31.5	35
6	46	Male	608	IV	35.9	<1	343.9	25
7	77	Female	162	SC	124	2.9	948.8	30

IV: intravenous, SC: subcutaneous, TCZ: tocilizumab, ACR: urinary albumin-to-creatinine ratio, GFR: glomerular filtration rate

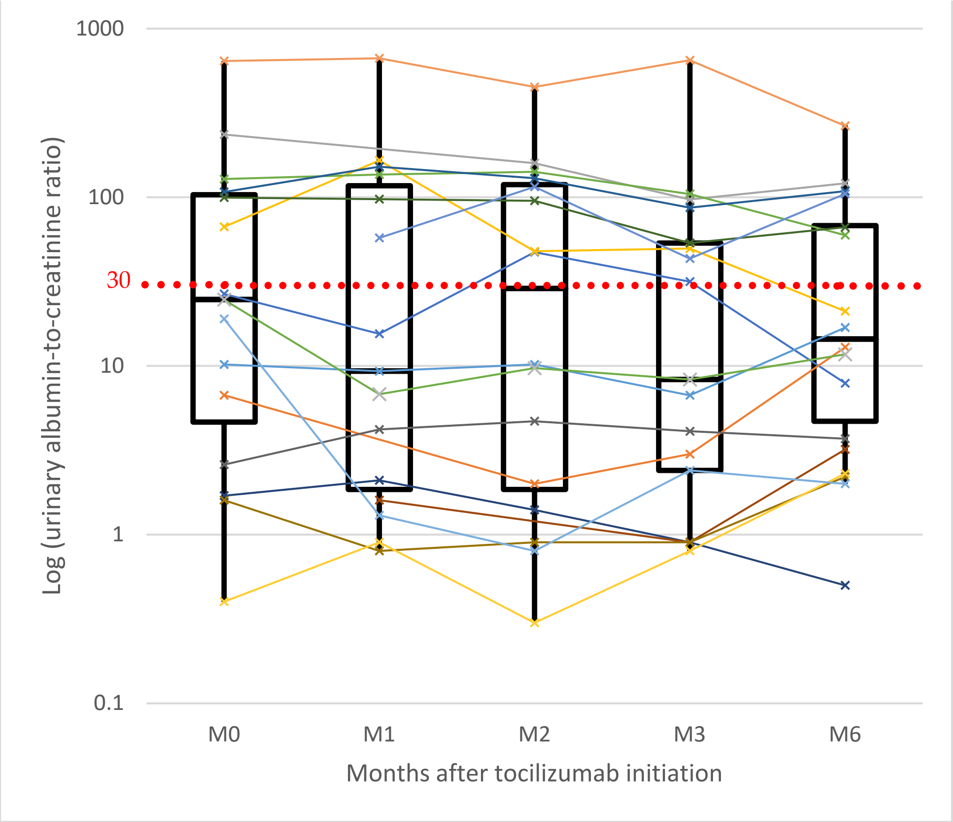

Figure S1. Temporal evolution of the urinary albumin-to-creatinine ratio (ACR) before tocilizumab administration (M0) and at M1, M2, M3 and M6 after tocilizumab administration. Each colored line represents one patient. Box plots represent the medians and 25th-75th percentile. The red dotted line represents the threshold value of macroalbuminuria (ACR = 30 mg/mmol). Friedman test, $X^2 = 3.83$, $p = 0.430$.
